# Comparative gonadotoxicity of the chemotherapy drugs cisplatin and carboplatin on prepubertal mouse gonads

**DOI:** 10.1093/molehr/gaaa008

**Published:** 2020-01-18

**Authors:** Caroline M Allen, Federica Lopes, Rod T Mitchell, Norah Spears

**Affiliations:** 1 Biomedical Sciences, University of Edinburgh, Edinburgh, EH8 9XD, UK; 2 MRC Centre for Reproductive Health, University of Edinburgh, Edinburgh, EH16 4TJ, UK; 3 Current Address: MRC Centre for Reproductive Health, University of Edinburgh, Edinburgh, EH16 4TJ, UK

**Keywords:** chemotherapy, fertility preservation, ovary, follicles, testis, spermatogonia, tissue culture, gonadotoxicity

## Abstract

The treatment of childhood cancer with chemotherapy drugs can result in infertility in adulthood. Newer generations of drugs are developed to replace parent drugs, with the potential benefits of less toxic side effects. For platinum alkylating-like drugs, in contrast to the parent compound cisplatin, the newer-generation drug carboplatin is reported to have reduced toxicity in some respects, despite being administered at 5–15 times higher than the cisplatin dose. Whether carboplatin is also less toxic than cisplatin to the reproductive system is unknown. Here we compare the gonadotoxic impact of cisplatin and carboplatin on female and male mouse prepubertal gonads. *In vitro* cultured CD1 mouse ovaries or testis fragments were exposed to either cisplatin or carboplatin for 24 h on Day 2 of culture and analysed by Day 6. A dose response for each drug was determined for the ovary (0.5, 1 & 5 μg/ml cisplatin and 1, 5 & 10 μg/ml carboplatin) and the testis (0.01, 0.05 & 0.1 μg/ml cisplatin and 0.1, 0.5 & 1 μg/ml carboplatin). For the ovary, unhealthy follicles were evident from 1 μg/ml cisplatin (73% unhealthy, *P* = 0.001) and 5 μg/ml carboplatin (84% unhealthy, *P* = 0.001), with a concomitant reduction in follicle number (*P* = 0.001). For the testis, the proliferating germ cell population was significantly reduced from 0.05 μg/ml cisplatin (73% reduction, *P* = 0.001) and 0.5 μg/ml carboplatin (75% reduction, *P* = 0.001), with no significant impact on the Sertoli cell population. Overall, results from this *in vitro* animal model study indicate that, at patient equivalent concentrations, carboplatin is no less gonadotoxic than cisplatin.

## Introduction

Childhood cancer survival rates have markedly increased in recent decades, with the current 5-year survival rate across all childhood cancers above 80% (Miller *et al.*, 2016). This improvement can be attributed in large part to the increasing use of chemotherapy drugs in treatment regimens since their introduction to clinical practice in the early 1940s (Devita and Chu, 2008), along with targeted therapies such as radiotherapy, immunotherapy and surgery. Multiple chemotherapy agents have been developed targeting malignant tumours through various mechanisms, with treatment most often combining different classes of drugs (Lind, 2011). However, damage to healthy tissues can arise as a side effect of chemotherapy treatment, since these cytotoxic drugs can target normal cells as well as malignant ones. It is becoming increasingly apparent that a vast majority of childhood cancer survivors are living with long-term chronic health conditions as a result of the treatment they received (Oeffinger *et al.*, 2006; Hudson *et al.*, 2013; Robison and Hudson, 2014; Bhakta *et al.*, 2017). The impact upon future fertility has been extensively studied, and there are now clear links between the use of alkylating chemotherapy agents in treatment regimens for childhood cancers and subsequent impairment of fertility (Anderson and Wallace, 2016; Stukenborg *et al.*, 2018; Newton *et al.*, 2019). Indeed, a study by Chow *et al*. (2016) has shown a reduced fertility rate for male and, to a lesser extent, female survivors of childhood cancer in comparison to their siblings. In short, it is clear that certain chemotherapy agents damage the reproductive systems of those children undergoing treatment. The most commonly used drugs have been broadly classified according to their predicted risk to future fertility, although these classifications are a matter of debate (Wallace *et al*., 2005; Wyns *et al*., 2010; Wallace, 2011). Many studies are based on historic treatment protocols that may be outdated and also more aggressive in comparison to current oncology regimens, with newer generations and combinations of drugs considered to be more effective with reduced side effects. As a result, more information is needed on these newer protocols and generations of drugs in order to determine the risk of subsequent infertility for today’s patients.

**Figure 1:**
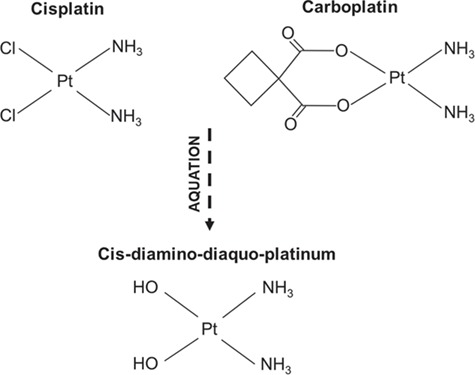
**Molecular structure of platinum-alkylating chemotherapy agents and active metabolites formed following aquation of parent drug.** Cisplatin and carboplatin share a similar chemical structure with a core platinum ion surrounded by two ‘amine groups’ and two ‘leaving groups’. Cisplatin and carboplatin differ in the leaving groups as shown: cisplatin is composed of chloride ion leaving groups whereas carboplatin contains cyclobutane-decarboxylate leaving groups. Once inside cells, the leaving groups are displaced by water forming aquated active metabolites. The chloride leaving groups of cisplatin readily dissociate whereas the cyclobutane-decarboxylate groups have reduced aquation rates requiring an esterase to dissociate from the parent compound. As a result, cisplatin and carboplatin have different rates of aquation which could account for the reduced clinical activity of carboplatin requiring higher doses to be administered to patients for the drug to be as effective as cisplatin (Ho *et al.*, 2016).

Platinum alkylating-like chemotherapy drugs are commonly used in paediatric oncology treatment regimens for multiple different types of cancer, including germ cell tumours, carcinomas, lymphomas and sarcomas (Dasari and Tchounwou, 2014). Development of these platinum-containing compounds has resulted in different generations including cisplatin (1st generation) and carboplatin (2nd generation) (Weiss and Christian, 1993). These drugs are composed of doubly charged platinum ions surrounded by four ligands: two amine groups and two ‘leaving groups’ (Desoize and Madoulet, 2002) (Fig. 1). Once inside the cell, the drugs are hydrolysed to form active metabolites that bind to and damage cellular DNA, RNA and proteins, resulting in activation of cell death pathways. Toxicity is mainly attributed to DNA damage where the active metabolites bind to purine bases resulting in cross-linking of the DNA (Dasari and Tchounwou, 2014). However, other molecular mechanisms have been linked to the clinical activity of these drugs, including induction of oxidative stress, modulation of calcium signalling and activation of cellular pathways (Dasari and Tchounwou, 2014). Cisplatin is associated with many toxic side effects including emesis, nephrotoxicity, neurotoxicity, myelosuppression, immunosuppression and ototoxicity; together, these side effects can limit the use of cisplatin in treatment protocols (Florea and Büsselberg, 2011). Cisplatin has been broadly classified as gonadotoxic for paediatric patients (Wyns *et al.*, 2010; Chow *et al.*, 2016), a categorisation that has been backed up by animal model studies (reviewed in Morgan *et al*., 2012; Allen *et al*., 2018). Newer generations of this class of drugs have been developed with reduced toxicity; so far, carboplatin is the main drug which has been widely and successfully used as a replacement of cisplatin, although only for certain cancers (Weiss and Christian, 1993; Go and Adjei, 1999). Pharmacologically, carboplatin is 8–45 times less potent than cisplatin for certain cancers and is more stable, possibly due to reduced aquation rates and DNA-damaging effects (Micetich *et al*., 1985; Knox *et al*., 1986; reviewed in: Ruggiero *et al*., 2013; Dasari and Tchounwou, 2014) (Fig. 1). As a consequence, clinically, carboplatin is administered at higher doses in order to be as effective as cisplatin (Hongo *et al.*, 1994; Dasari and Tchounwou, 2014). From published paediatric patient doses, the administered dose of carboplatin can range from 5–15 times higher than the administered dose of cisplatin (Crom *et al*., 1981; Dominici *et al*., 1989; Madden *et al*., 1992; Murry *et al*., 1993; Newell *et al*., 1993; Riccardi *et al*., 1994; Peng *et al*., 1997; Erdlenbruch *et al*., 2001; Clerico *et al*., 2006; Veal *et al*., 2007). So far, few studies have focused upon the toxicity of newer generations of platinum alkylating-like agents, although carboplatin has been reported to reduce damage to the kidneys in comparison to cisplatin (Canetta *et al.*, 1985). However, myelosuppression and emesis remain associated side effects of both drugs (Canetta *et al.*, 1985). As with cisplatin, carboplatin is currently considered as gonadotoxic based on current classification systems (Wallace *et al.*, 2005; Wyns *et al.*, 2010; Lambertini *et al.*, 2016), although to the best of the authors’ knowledge, no studies have focused specifically on the gonadotoxicity of carboplatin in paediatric patients or in prepubertal animal models.

This study used cultured prepubertal ovaries and testes to compare the gonadotoxicity of carboplatin and cisplatin. *In vitro* systems are widely used to test and compare different drugs in reproductive toxicology (Stefansdottir *et al.*, 2014), and the methods used here are well-established models for culturing immature mouse gonadal tissue, supporting the health and physiological development of control cultured tissue (see for example Morgan *et al.*, 2013; Smart *et al.*, 2018). Whereas whole ovaries can remain healthy when cultured intact, the size of the testes results in large areas of necrosis if cultured whole, and so testicular tissue was dissected into small fragments prior to culture as carried out previously by ourselves and others (see for example Sato *et al.*, 2011; Lopes *et al.*, 2016; Smart *et al.*, 2018). To compare the two drugs in both the ovary and the testis, a dose response for each drug was first obtained in order to determine a lowest dose that had no effect on that tissue and a highest dose that induced maximal damage, resulting in few, if any, healthy follicles in the ovary or surviving germ cells in the testis. The resulting dose response concentrations fall within clinical patient serum levels for each drug, with tissues exposed to higher concentrations of carboplatin than cisplatin, reflecting the relative dose administered to patients (Ruggiero *et al.*, 2013).

## Methods

### Animals

Experiments were approved by the University of Edinburgh’s Local Ethical Review Committee and carried out in accordance with UK Home Office regulations. CD1 mice were housed in an approved animal facility and kept in a 14-hr light:10-hr dark cycle, with food and drink provided *ad libitum*. Animals were sacrificed through cervical dislocation.

### Chemotherapy drugs

Cisplatin and carboplatin (Millipore, UK) were purchased in powder form, made up in distilled water to stock solutions of 1 mg/ml cisplatin and 10 mg/ml carboplatin. The chemotherapy drugs were added to the culture medium to produce final concentrations that resulted in a dose response for each tissue that ranged from a lowest dose that had no significant effect to a highest dose that affected the health (ovary) or number (testis) of the vast majority of germ cells. The dose response was based upon the health/number of the follicles/germ cells at the end of the culture period. For the ovary, concentrations of 0.5, 1 and 5 μg/ml cisplatin and 1, 5 and 10 μg/ml carboplatin were added; for the testis, lower concentrations of 0.01, 0.05 and 0.1 μg/ml cisplatin and 0.1, 0.5 and 1 μg/ml carboplatin were sufficient to produce the required dose response: all doses used were broadly within the range that has been found in patient serum, as detailed below.

### Culture systems

Prepubertal mouse ovary and testis culture systems were performed as previously described and validated (Lopes *et al*., 2016; Smart *et al*., 2018), using hormone- and antibiotic-free culture conditions. Briefly, ovaries (postnatal day 4; pnd4) and testes (pnd5) were dissected and placed in Leibovitz L-15 medium (Invitrogen, UK) supplemented with 3 mg/ml bovine serum albumin (BSA; Sigma, UK) prewarmed to 37°C.

#### Ovary culture

For each experimental run, ovaries were dissected from all females within a litter, with the ovaries then randomly distributed across treatment groups. Non-gonadal tissues were removed, and each ovary was cultured upon a floating polycarbonate membrane (Whatman Nuclepore Polycarbonate Membrane, Camlab Ltd, Cambridge, UK) in a 24-well plate (Greiner Bio-One, Stonehouse, UK) containing 1 ml α-minimum essential media (α-MEM, Invitrogen, UK) supplemented with 3 mg/ml fatty acid free BSA (Sigma, UK) and incubated at 37°C with 5% CO_2_ (Day 1). Following 24 h of chemotherapy exposure (Day 2), tissues were removed and placed in a drug-free medium until the end of culture (Days 3–6), with media changes every 2/3 days. Vehicle control cultures containing drug-free media supplemented with water in place of drugs were maintained throughout the culture period. Tissues were fixed in Bouin’s (Sigma, UK) for morphological analysis. For all groups, six ovaries were analysed (*n* = 6), obtained from at least four separate litters.

#### Testis culture

For each culture experiment, testes were obtained from two or three pups from the same litter. Non-gonadal tissues and surrounding tunica were removed, and testes were dissected into roughly 0.1-mm^3^ fragments using a blade (Altomed, UK), with the testicular fragments then randomly distributed across treatment groups. Each fragment was cultured on a floating membrane within a 24-well plate containing 1 ml α-MEM media supplemented with 10% knockout serum replacement (Invitrogen, UK). Tissues were cultured at 37°C in 5% CO_2_. After a settling in period of 24 h (Day 1), tissues were exposed to drugs for 24 h (Day 2), after which the floating membranes were moved into drug-free media (Day 3). To determine the proliferative capacity of tissues, 15 μg/ml of bromodeoxyuridine (BrdU; Sigma, UK) was added for the final 24 h of culture (Day 4). Vehicle control cultures containing drug-free media supplemented with water were maintained throughout the culture period. At end of the culture period, tissues were fixed in 10% neutral buffered formalin (NBF; Sigma, UK) for immunohistochemistry. For all groups, six fragments were analysed (*n* = 6), obtained from at least three separate litters.

### Morphological evaluation of ovary

**Figure 2:**
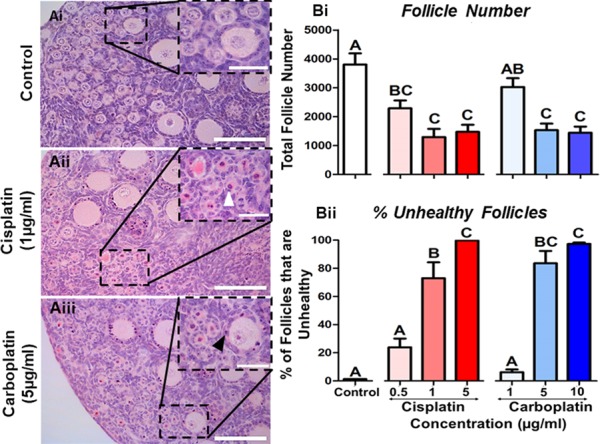
**Follicle number and health are reduced following cisplatin or carboplatin treatment. (A)** Representative histological sections of (Ai) control, (Aii) cisplatin (1 μg/ml) and (Aiii) carboplatin (5 μg/ml) exposed tissue; insets show higher magnification of framed areas. White arrowhead shows example of a damaged oocyte; black arrowhead shows example of a damaged granulosa cell; scale bars represent 50 μm, or 20 μm for insets. (**B**) Histograms show (Bi) total follicle number and (Bii) the percentage of unhealthy follicles following exposure to cisplatin (red) or carboplatin (blue); *n* = 6 for all experimental conditions. Data are mean + SEM; columns without letters in common are statistically significantly different to each other (*P* < 0.05).

Fixed ovaries were embedded in paraffin and 5-μm sections cut. To evaluate morphology, follicle number, class and health were determined as in Morgan *et al*. (2013). Every sixth section was analysed, with total follicle counts estimated using the Abercrombie formula (Abercrombie, 1946). Tissues were stained with haematoxylin and eosin (H&E); images were taken with a DMLB Leica microscope (Leica Microsystems Ltd, UK) and analysed using ImageJ software (JAVA) with the assessor blind to experimental conditions. Follicles were included in the count only where germinal vesicles were visible. Follicle stage and health were determined based on appearance of granulosa cells and oocytes as described in Morgan *et al*. (2013). Follicles were classed as either primordial, transitional or primary, with overall health determined using standard morphological criteria as either unhealthy due to damage to the oocytes (Fig. 2 Aii, white arrowhead), to the granulosa cells (Fig. 2 Aiii, black arrowhead) or to both cell types. Oocyte damage was characterised based on the appearance of shrunken, eosinophilic cytoplasm or condensed nuclear chromatin, while granulosa cell damage was evidenced by the condensed chromatin or irregular overall shape of at least one granulosa cell, as detailed in previous work by ourselves and others (see for example Springer *et al.*, 1996; Muruvi *et al.*, 2005; Morgan *et al.*, 2013; Lopes *et al.*, 2014, 2016).

### Immunohistochemistry of testis

Fixed testis fragments were embedded in paraffin and serially sectioned at 5 μm. Every fifth section was immunostained. In brief, slides were dewaxed, rehydrated and subjected to antigen retrieval with citrate buffer (10 mM sodium citrate, pH 6, Fisher Chemical, UK). Slides were blocked in 20% goat serum in PBS (Thermo Fisher Ltd, UK) with 0.1% Triton X-100 (PBST; Sigma, UK) and 5% BSA for 1 h. Primary antibodies were incubated overnight at 4°C and secondary antibodies for 1 h at room temperature in 10% goat serum, PBST and 5% BSA; see Table I for all antibody details. Slides were counterstained with DAPI (Invitrogen, UK) and mounted with Vectashield mounting medium (Vector Laboratories, USA). Images were taken (Leica DM4400B microscope on a DFC360FX camera) and analysed using ImageJ (JAVA) with the assessor blind to experimental conditions. The density of total proliferating germ cells (MVH-positive/BrdU-positive), non-proliferating germ cells (MVH-positive/BrdU-negative) and proliferating Sertoli cells (SOX9-positive/BrdU-positive) were determined through manual counting per area of seminiferous tubule. Area of fluorophore expression for Sertoli cells (SOX9-positive) per area of seminiferous tubules was used to determine density of Sertoli cells as described in Smart *et al*. (2018).

**Table I:** **Primary and secondary antibodies for double immunohistochemistry**.

*Antigen 1*	*Dilution*	*Secondary Antibody for Antigen 1*	*Antigen 2*	*Dilution*	*Secondary Antibody for Antigen 2*
Mvh (ab27591)	1:200	GAMou-488	BrdU (ab6326)	1:500	GARat-568
Sox9 (ab5535)	1:500	GARab-488	BrdU (ab6326)	1:500	GARat-568

Primary antibodies (antigen 1 and 2) purchased from Abcam, UK, and Secondary antibodies purchased from Invitrogen, UK.

**Figure 3:**
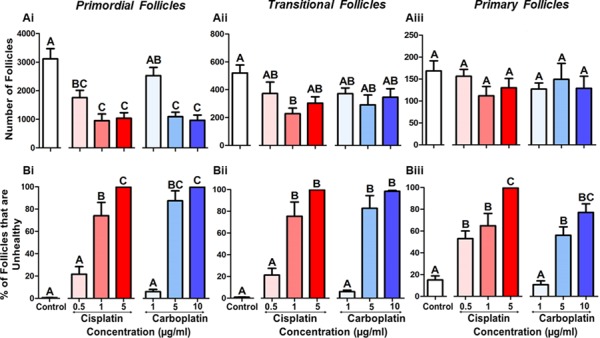
**Cisplatin or carboplatin treatment reduces the number of primordial follicles while damaging the health of all follicles. (A)** number and (**B**) percentage of follicles classified as unhealthy for (i) primordial, (ii) transitional or (iii) primary follicles following cisplatin (red) or carboplatin (blue) treatment, *n* = 6 for all experimental conditions. Data are mean + SEM; columns without letters in common are statistically significantly different to each other (*P* < 0.05).

### Statistical analysis

Statistical analysis was performed using Minitab statistical software programme version 17 (Minitab Inc., State College, PA, USA), and graphs were created using GraphPad Prism software version 5 (GraphPad Software, Inc., CA, USA). A one-way ANOVA with general linear mixed effect model was used to determine statistical significance of treatment, with effect of culture run taken into account; results were deemed statistically significant at *P* < 0.05. Tukey pairwise comparison test determined statistical significance between the experimental conditions. Histograms of results are represented as mean + SEM. Significance between groups is symbolised on graphs by letters, with different letters indicating a statistical significance difference with individual *P* values of *P* < 0.05.

## Results

### Cisplatin or carboplatin exposure reduces the number and health of follicles in the prepubertal ovary

In order to assess the effects of cisplatin or carboplatin treatment, ovaries were examined after exposure *in vitro* to a range of concentrations that gave a dose response (cisplatin—0.5, 1 and 5 μg/ml; carboplatin—1, 5 and 10 μg/ml; Fig. 2A). Total follicle number was reduced compared to controls following exposure to all concentrations of cisplatin, with a reduction from 0.5 μg/ml (40%; *P* = 0.007), 1 μg/ml (66%; *P* = 0.001) and 5 μg/ml (61%; *P* = 0.001) and from 5 μg/ml (60%; *P* = 0.001) and 10 μg/ml (62%; *P* = 0.001) of carboplatin (Fig. 2 Bi). The percentage of unhealthy follicles increased in a dose-dependent manner when exposed to at least 1 μg/ml of cisplatin (73 + 11% unhealthy; *P* = 0.001) and 5 μg/ml carboplatin (84 + 9% unhealthy; *P* = 0.001) in comparison to the control (Fig. 2 Bii). At the highest concentrations, almost all follicles were unhealthy at 5 μg/ml cisplatin (100% unhealthy; *P* = 0.001) and 10 μg/ml carboplatin (97 + 1% unhealthy; *P* = 0.001).

### Cisplatin or carboplatin targets follicles independent of stage

Since the number and health of the follicles were reduced following treatment with either drug, the follicles were further classified as primordial, transitional or primary to determine if a particular follicle class was targeted. Both cisplatin and carboplatin reduced the number of primordial follicles in comparison to the control from 0.5 μg/ml cisplatin (44%; *P* = 0.004) and 5 μg/ml carboplatin (65%; *P* = 0.001) (Fig. 3 Ai). There was little effect of cisplatin or carboplatin treatment on the number of growing transitional or primary follicles, although a trend towards a decrease in transitional follicles was noted, with a significant reduction in number at 1 μg/ml (56%; *P* = 0.029) of cisplatin only (Fig. 3 Aii,iii). When follicle health rather than number was examined, the percentage of unhealthy follicles increased for all classes in a dose-dependent manner. Damage to the primordial follicles was evident from 1 μg/ml cisplatin (74 + 12% unhealthy; *P* = 0.001) and 5 μg/ml carboplatin (87 + 9% unhealthy; *P* = 0.001) (Fig. 3 Bi). A significant number of unhealthy transitional follicles was observed from 1 μg/ml cisplatin (75 + 13% unhealthy; *P* = 0.001) and 5 μg/ml carboplatin (83 + 12% unhealthy; *P* = 0.001) (Fig. 3 Bii). For the primary follicles, damage was seen from 0.5 μg/ml cisplatin (53 + 7% unhealthy; *P* = 0.005) and 5 μg/ml carboplatin (56 + 8% unhealthy; *P* = 0.002) (Fig. 3 Biii).

### Granulosa cells of primary follicles are damaged by cisplatin or carboplatin exposure

**Figure 4:**
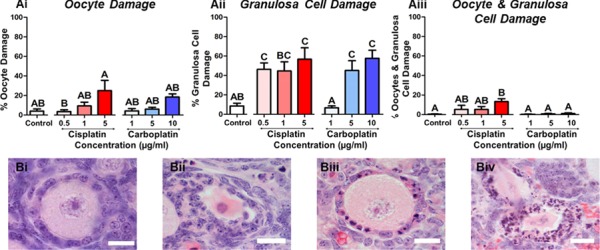
**Granulosa cells of the primary follicles are damaged by cisplatin or carboplatin.** Percentage of primary follicles classed as unhealthy due to (Ai) oocyte, (Aii) granulosa cell or (Aiii) combined (oocyte and granulosa cell) damage following cisplatin (red) or carboplatin (blue) treatment, *n* = 6 for all experimental conditions. Data are mean + SEM; columns without letters in common are statistically significantly different to each other (*P* < 0.05). Representative images show (Bi) healthy primary follicle (Bii) primary follicle with damaged oocyte, (Biii) primary follicle with damaged granulosa cells and (Biv) primary follicles with combined (oocyte and granulosa cell) damage. Scale bar = 10 μm.

The health of each primary follicle was assessed depending upon whether the oocyte and/or granulosa cells were damaged following cisplatin or carboplatin exposure (Fig. 4). This analysis was not carried out for primordial or transitional follicles due to the difficulty of properly assessing the health of flattened granulosa cells. In primary follicles, either cisplatin or carboplatin treatment damaged the granulosa cells in comparison to control. These effects were seen from 0.5 μg/ml cisplatin (46 + 7% unhealthy; *P* = 0.037) and from 5 μg/ml carboplatin (45 + 10% unhealthy; *P* = 0.049) (Fig. 4 Aii).

### Cisplatin or carboplatin exposure reduces the proliferating germ cell population in the prepubertal testis

**Figure 5:**
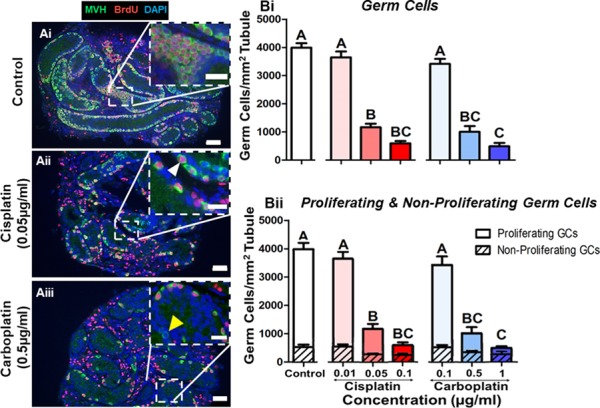
**Cisplatin or carboplatin treatment results in a significant loss of the proliferating germ cells.** (**A**) Representative images of histological sections from (Ai) control, (Aii) cisplatin (0.05 μg/ml) and (Aiii) carboplatin (0.5 μg/ml) treated testis, showing a reduction in the density of proliferating germ cells (MVH-positive, green; BrdU-positive, red). Insets show higher magnification of framed areas. White arrowhead shows example of a proliferating germ cell (MVH-positive/BrdU-positive); yellow arrowhead shows example of a non-proliferating germ cell (MVH-positive/BrdU-negative). Scale bars represent 50 μm, or 25 μm for insets. (**B**) Density of germ cells per seminiferous tubule area (mm^2^) following cisplatin (red) or carboplatin (blue) treatment. Data shows the density of the (Bi) total, (Bii: open bars) proliferating and (Bii: hatched bar) non-proliferating germ cells; *n* = 6 for all experimental conditions. Data are mean + SEM; columns without letters in common are statistically significantly different to each other (*P* < 0.05). For (Bii), letters refer to proliferating germ cells (open bars) only, with no statistically significant difference between groups for the non-proliferating germ cells (hatched bars).

To determine the impact of cisplatin or carboplatin treatment on the prepubertal testis, testicular fragments were exposed to cisplatin (0.01, 0.05 and 0.1 μg/ml) or carboplatin (0.1, 0.5 and 1 μg/ml) *in vitro* (Fig. 5 A). Germ cells (MVH-positive) were counted and classified as proliferating (MVH-positive/BrdU-positive) or non-proliferating (MVH-positive/BrdU-negative). The density of the total germ cell population was significantly reduced in a dose-dependent manner evident from 0.05 μg/ml cisplatin (71% decrease; *P* = 0.001) and 0.5 μg/ml carboplatin (75% decrease; *P* = 0.001) (Fig. 5 Bi). A low density of germ cells remained after treatment at the highest concentrations of both drugs in comparison to control with 15% of germ cells remaining after cisplatin (0.1 μg/ml; *P* = 0.001) and 12% after carboplatin (1 μg/ml; *P* = 0.001) treatment. Cisplatin and carboplatin both affected the proliferating germ cell population (MVH-positive/BrdU-positive), with the density significantly decreased in a dose-dependent manner following exposure to at least 0.05 μg/ml cisplatin (73% decrease; *P* = 0.001) and 0.5 μg/ml carboplatin (75% decrease; *P* = 0.001) (Fig. 5 Bii). A small population of non-proliferating germ cells (MVH-positive/BrdU-negative) was observed in the control tissue, with a similar density (~400 + 30 cells per mm^2^ of tubule) of these cells maintained across all experimental groups.

### Sertoli cell density is unaffected by either cisplatin or carboplatin exposure

**Figure 6:**
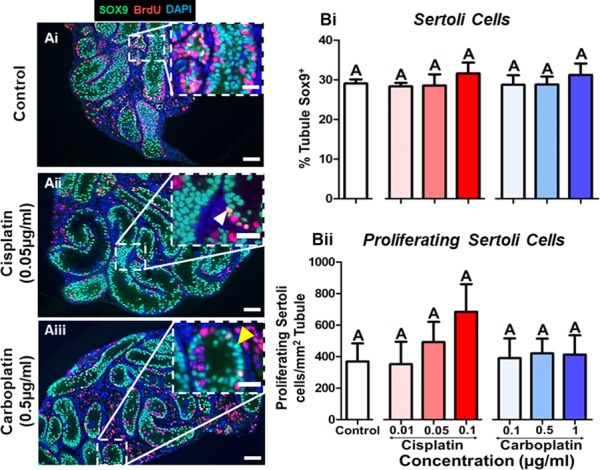
**Density and proliferation of Sertoli cells are not affected by cisplatin or carboplatin. (A)** Representative images of (Ai) control, (Aii) cisplatin (0.05 μg/ml) and (Aiii) carboplatin (0.5 μg/ml) treated tissues showing no change in density of Sertoli cells (SOX9-positive; green) or in proliferating Sertoli cells (SOX9-positive/BrdU-positive; orange). Insets show higher magnification of framed areas. White arrowhead shows example of a proliferating Sertoli cell; yellow arrowhead shows example of a non-proliferating Sertoli cell; scale bars represent 50 μm, or 25 μm for insets. (**B**) Data shows (Bi) density of Sertoli cells measured by the % area of the seminiferous tubules expressing Sox9, following cisplatin (red) or carboplatin (blue) treatment as well as (Bii) number of manually counted proliferating Sertoli cells. *n* = 6 for all experimental conditions. Data are mean + SEM; columns with different letters are statistically significantly different to each other (*P* < 0.05).

To assess whether the damaging effect of cisplatin or carboplatin exposure on germ cell density was also seen on the somatic cells present within the seminiferous tubules, we determined the overall density of Sertoli cells (SOX9-positive) (Fig. 6 A). This was unaffected by cisplatin or carboplatin exposure (*P* = 0.641) (Fig. 6 Bi). Similarly, the proliferative capacity of these cells (SOX9-positive/BrdU-positive) were also not altered by exposure to either drug (*P* = 0.283) (Fig. 6 Bii).

## Discussion

Prepubertal mouse ovaries and testes were exposed to a range of concentrations of either cisplatin or carboplatin in culture, within patient relevant concentrations; both drugs induced a dose-dependent response. In order to achieve a similar clinical efficacy, carboplatin must be administered at concentrations 5–15 times higher than cisplatin (Hongo *et al.*, 1994; Dasari and Tchounwou, 2014). Here, results show that for ovarian tissue, two to five times carboplatin concentrations (1, 5 and 10 μg/ml) in comparison to cisplatin (0.5, 1 and 5 μg/ml) induced equivalent levels of damage to the follicles, which resulted in significant effects at or above 1 μg/ml cisplatin and 5 μg/ml carboplatin in terms of health. For the testicular tissue, 10 times carboplatin doses (0.1, 0.5 and 1 μg/ml) were required to induce a similar reduction in germ cell density compared to cisplatin doses (0.01, 0.05 and 0.1 μg/ml), resulting in significant effects at or above 0.05 μg/ml cisplatin and 0.5 μg/ml carboplatin.

This is the first study to compare the effects of cisplatin or carboplatin exposure on the prepubertal gonad in females and males. Several clinical studies in adults have compared the efficacy, survival and toxicities of these drugs in treating a range of cancers where cisplatin has, in general, better survival rates and efficacy while carboplatin has a better toxicity profile; however, this is not the case for all malignancies (reviewed in Ho *et al.*, 2016). For paediatric patients, carboplatin has been found to be as effective as cisplatin in treating germ cell tumours with the added benefit of a reduced toxicity profile (Ruggiero *et al.*, 2013; Frazier *et al.*, 2018; Shah *et al.*, 2018). The toxicity of these platinum alkylating-like drugs is mainly attributed to the formation of DNA adducts, resulting in cross-linkage and subsequent DNA damage: whether there is any underlying mechanism that may account for the observed differences in toxicity prolife of these two drugs is, however, not yet clear (reviewed in Siddik, 2003; Dasari and Tchounwou, 2014). One study in cancer cell lines has identified a potential difference between the two drugs, with cisplatin producing higher levels of oxidative stress than carboplatin at patient-equivalent concentrations (Marullo *et al.*, 2013); it might be of interest to explore the oxidative stress response to cisplatin and carboplatin in the gonads in future studies. Our study compares the effects of these drugs on prepubertal gonadal tissues and found, relative to the concentrations of each drug in patient serum, gonadotoxicity resulting from carboplatin exposure was at least as bad as that resulting from cisplatin exposure (patient serum levels: cisplatin—0.05-4 μg/ml; Crom *et al.* 1981; Dominici *et al.* 1989; Peng *et al.* 1997; Erdlenbruch *et al.* 2001 and carboplatin—0.5–90 μg/ml; Madden *et al.*, 1992; Murry *et al.*, 1993; Newell *et al.*, 1993; Riccardi *et al.*, 1994; Clerico *et al.*, 2006; Veal *et al.*, 2007). This is of particular importance, as carboplatin as a second-generation drug of the platinum alkylating-like class was developed as an alternative to cisplatin in part due to reduced nephrotoxicity, ototoxicity and emesis for paediatric patients (reviewed in Ruggiero *et al.*, 2013). Overall, carboplatin doses of two to five times for the ovary and 10 times for the testis were found to be as damaging as equivalent cisplatin concentrations. The same conclusion was also reached in an *in vivo* study of adult mice based on the morphology of the testis, where 10 times carboplatin was found to be as gonadotoxic as cisplatin (Köpf-Maier, 1992). Reduced spermatogenesis and altered structure of the seminiferous tubules were observed, as well as decreased integrity of Sertoli cells disrupting tight junction contact, a factor that was not investigated in our study. Another such study of adult males found carboplatin to be less toxic than cisplatin with regard to steroidogenesis (Azouri *et al.*, 1989): our study did not investigate the impact on steroidogenesis. Our data from this *in vitro*, animal model study suggests that replacing cisplatin with carboplatin in treatment regimens will not prevent or reduce chemotherapy-induced gonadotoxicity. Based on our short-term study, carboplatin may be even more damaging to the ovary than cisplatin, since only two to five times the carboplatin concentration induced equivalent damage as the cisplatin, whereas patients can receive up to 15 times the dose. It will be important for further studies to focus on clinical paediatric patients, to determine whether the same conclusions can be drawn on future fertility as from this short-term animal study.

For both cisplatin and carboplatin, higher doses were required to observe significant levels of germ cell/follicle damage/loss to the ovarian tissue in comparison to the testes, suggesting the testis may be more sensitive to the platinum alkylating-like agents. This has previously been noted for other chemotherapy drugs including irinotecan (Lopes *et al.*, 2016), although that could, in part at least, be due to differences in the culture conditions of ovarian and testicular tissue. It is also important to bear in mind that, whereas there would be the potential for even a few surviving spermatogonial stem cells to (partially) repopulate the testis after treatment, any loss of oocytes would be irreplaceable. Nonetheless, in agreement with our current and previous (Lopes *et al.*, 2016) findings that the testis may be more sensitive to chemotherapy treatment than the ovary, a study following survivors of childhood cancer that had been treated with alkylating agents found that the effects on male fertility were more pronounced than those on female fertility (Chow *et al.*, 2016); the decrease in the percentage of male survivors siring a live birth compared to their siblings was greater than the decrease in the percentage of female survivors having live births compared to their siblings. Additional studies are required to determine the functional deficits following cisplatin or carboplatin treatment and the long-term impact on recovery and subsequent effects on fertility.

Ovarian toxicity as a result of chemotherapy treatment can lead to infertility and premature ovarian insufficiency (POI) due to direct damage to the ovary depleting the ovarian reserve of primordial follicles (reviewed in Ben-Aharon and Shalgi, 2012; Morgan *et al.*, 2012; Bedoschi *et al.*, 2016; Spears *et al.*, 2019). Primordial follicles form before birth when the germ cells leave the mitotic phase, enter meiosis, arrest at prophase I and become surrounded by pregranulosa cells. These primordial follicles represent a female’s reproductive potential and are activated into growing follicles throughout the reproductive lifespan of the female (McGee and Hsueh, 2000). Loss of primordial follicles, through for example chemotherapy treatment, can lead to POI and early menopause (Wallace *et al.*, 2005). Our study shows that cisplatin and carboplatin exposure leads to a reduction in the number of primordial follicles aligning with previous studies on cisplatin (Meirow, 2000; Gonfloni *et al.*, 2009; Morgan *et al.*, 2013). All follicles, including those at the primordial state, were damaged in our study, showing that direct effects of the drugs on the primordial follicles is at least part of the reason for the primordial follicle loss. Overall, primordial follicle loss may be a result of either direct damage to the primordial follicles and/or accelerated activation of primordial follicles into the growth phase as a result of the drugs depleting the growing follicular pool that usually inhibits activation (Meirow *et al.*, 2010; Findlay *et al.*, 2015). In the literature, the underlying mechanism for primordial follicle loss following exposure to platinum alkylating-like agents is still debated, with some studies showing direct damage to the primordial follicles (Nguyen *et al.*, 2019) and others suggesting that accelerated activation contributes to loss of primordial follicles following cisplatin treatment (Morgan *et al.*, 2013; Chang *et al.*, 2015).

The specific cell type within primary ovarian follicles that was affected by cisplatin or carboplatin treatment was further investigated, to determine whether the oocyte and/or granulosa cells were targeted. The oocyte and surrounding granulosa cells are co-dependent upon each other for oocyte maturation and folliculogenesis, therefore damage to one cell type will ultimately result in death of the latter, but it is also important to know which cell type is affected first if protective treatments are to be developed. Our results show that for primary follicles, both cisplatin and carboplatin primarily damage granulosa cells. Previous studies have focused on the oocyte or have combined primary follicles with transitional follicles to represent the growing follicular pool (Gonfloni *et al.*, 2009; Morgan *et al.*, 2013). These studies have shown effects of cisplatin on the oocyte, as a result of DNA damage, accumulation of Tap63 and activation of apoptotic pathways (Gonfloni *et al.*, 2009; Kerr *et al.*, 2012; Kim *et al.*, 2013, 2019; Morgan *et al.*, 2013; Rossi *et al.*, 2017; Nguyen *et al.*, 2018, 2019). These studies came to the conclusion that cisplatin targeted the oocyte, whereas our study found that for primary follicles, mainly granulosa cells were damaged following cisplatin treatment. The difference between these findings and the results of the present study is not clear, but could in part, be due to differences in mouse line (here CD1 line) or age of animal (here, pnd 4). The mechanism underlying granulosa cell damage following treatment with platinum containing alkylating-like agents has yet to be investigated fully.

For the testis, there is much less known about the effects of chemotherapy treatment on the prepubertal gonad than that of the adult. The paucity of prepubertal studies could possibly be due to limited animal model systems to investigate prepubertal testis development and/or to the fact that the prepubertal testis was once considered relatively inactive and therefore thought to be protected from any chemotherapy-induced damage (Rivkees and Crawford, 1988; Rey, 1999). The testis undergoes important developmental processes during prepuberty, including germ cell proliferation and Sertoli cell functional maturation, any of which could be affected by chemotherapy drugs (Chemes, 2001; Allen *et al.*, 2018). The present study shows that both cisplatin and carboplatin reduce the population of proliferating germ cells in the prepubertal testis. The proliferative capacity was analysed during a 24-h period starting 24 h after drug exposure had finished; therefore, the loss of proliferating cells could either be a result of the drugs targeting and killing the proliferating cells or a consequence of the drugs preventing subsequent germ cell proliferation. The underlying mechanism of action of the platinum alkylating-like agents has been studied following cisplatin treatment and indicates that cisplatin induces DNA damage and activates apoptotic pathways as shown through increased expression of cleaved caspases 3, TUNEL-positive cells and γH2AX expression (Lirdi *et al.*, 2008; Favareto *et al.*, 2011; Smart *et al.*, 2018). The germ cell population at this stage of development is predominantly composed of undifferentiated type A spermatogonia (spermatogonial stem cell; SSC) as well as differentiating spermatogonia type B (de Rooij and Russell, 2000; Ehmcke *et al.*, 2006). Further investigations should therefore focus on the specific germ cell types targeted, since our study used a generic marker for germ cells, MVH. Research is focusing on SSCs, since maintenance of this population is crucial for later fertility (Zohni *et al.*, 2012; Zohni *et al.*, 2012). SSCs are vulnerable to cisplatin treatment, with survival of isolated SSCs decreased in culture (Marcon *et al.*, 2010; Liu *et al.*, 2014; Shabani *et al.*, 2016) and PLZF-positive germ cells (SSCs) reduced in cultured testis tissue following cisplatin exposure (Smart *et al.*, 2018).

The density of the Sertoli cell population was unaffected by either cisplatin or carboplatin exposure as previously shown in Smart *et al.* (2018). However, there is the potential that functional maturation of these cells could be affected, resulting in Sertoli cells that are not capable of fully supporting germ cells through spermatogenesis in the adult (Chemes, 2001; Sharpe *et al.*, 2003). Indeed, previous studies have found a decrease in Sertoli cell viability (Aslani *et al.*, 2017) following treatment as well as functional deficits through reduced androgen binding protein or transferrin production (Nambu *et al.*, 1995; Favareto *et al.*, 2011). Here, additional studies would be required to investigate the maturation of these cells, through for example analysis of anti-Müllerian hormone and inhibin B (Dere *et al.*, 2013). In addition to the Sertoli cells, other somatic cells within the testis, including the endocrine Leydig cells and peritubular myoid cells, could be affected by chemotherapy treatment but have yet to be fully investigated. Initial studies using a mouse model found no impact of cisplatin on the density of the Leydig cell population (Smart *et al.*, 2018); however, there have yet to be any studies focusing on steroidogenesis or testosterone production following prepubertal exposure to either cisplatin or carboplatin.

In summary, working within the range of drug concentrations found in patient serum, results here show that clinically relevant concentrations of cisplatin and carboplatin have similar gonadotoxic effects on the prepubertal mouse ovary and testis. Both platinum-alkylating agents reduced the number and health of ovarian follicles. In the testis, either drug reduced the density of the proliferating germ cell population, while no effect on the density of Sertoli cells was seen. Since both drugs had an impact on gonadal tissues within clinically relevant concentrations, we conclude from this *in vitro* animal model study that replacing cisplatin with carboplatin in treatment protocols may not result in any additional protection of the gonadal tissues from the damaging effects of the drugs. Further clinical studies are required before any recommendations for paediatric patients can be made.
